# Scaling of Early Social Cognitive Skills in Typically Developing Infants and Children with Autism Spectrum Disorder

**DOI:** 10.1007/s10803-020-04449-9

**Published:** 2020-03-18

**Authors:** Katherine Ellis, Philippa Lewington, Laurie Powis, Chris Oliver, Jane Waite, Mary Heald, Ian Apperly, Priya Sandhu, Hayley Crawford

**Affiliations:** 1 Present Address: School of Psychology, University of Surrey, Guildford, Surrey GU2 7XH UK; 2 Cerebra Centre for Neurodevelopmental Disorders, School of Psychology, University of Birmingham, Birmingham, B15 2TT UK; 3 Present Address: The West Community Assessment and Treatment Service, Marlowes Health and Wellbeing Centre, 39-41 The Marlowes, Hemel Hempstead, HP1 1LD UK; 4 School of Life & Health Sciences, Aston University, Birmingham, B4 7ET UK; 5 School of Psychology, University of Birmingham, Birmingham, B15 2TT UK; 6 Present Address: CMHWR and Mental Health and Wellbeing Unit, Division of Health Sciences, Warwick Medical School, University of Warwick, Coventry, CV4 7AL UK; 7 Faculty of Health & Life Sciences, Coventry University, Coventry, UK

**Keywords:** Autism spectrum disorder, Theory of mind, Precursors, Social cognition

## Abstract

We delineate the sequence that typically developing infants pass tasks that assess different early social cognitive skills considered precursors to theory-of-mind abilities. We compared this normative sequence to performance on these tasks in a group of autistic (AUT) children. 86 infants were administered seven tasks assessing *intention reading* and *shared intentionality* (Study 1). Infants responses followed a consistent developmental sequence, forming a four-stage scale. These tasks were administered to 21 AUT children (Study 2), who passed tasks in the same sequence. However, performance on tasks that required following others’ eye gaze and cooperating with others was delayed. Findings indicate that earlier-developing skills provide a foundation for later-developing skills, and difficulties in acquiring some early social cognitive skills in AUT children.

**Electronic supplementary material:**

The online version of this article (10.1007/s10803-020-04449-9) contains supplementary material, which is available to authorized users.

‘Theory of Mind’ (ToM) is hypothesised to play a crucial role in successful interaction, allowing individuals to take the perspective of others and engage in complex social behaviour (Baron-Cohen et al. 2013). Evidence suggests that some ToM deficits might underpin difficulties in social interaction and communication in those with neurodevelopmental disorders, such as autistic children (AUT) (Baron-Cohen 1995; Baron-Cohen et al. 1985; Kimhi 2014), and genetic syndromes such as fragile X (Morel et al. 2018; Losh et al. 2012), Williams (Morel et al. 2018) and Down (Cebula et al. 2010) syndromes. ToM has been studied most intensively in young children aged 3 to 6 years (Wimmer and Perner 1983; Wellman et al. 2001), generating evidence that suggests ToM abilities in this period form a strict developmental sequence. However, little is known about the development of *early* social cognitive skills, which might act as precursors to ToM (Tomasello 2014; Wellman 2014). In study 1 we apply a scaling approach to outline the developmental sequence of *early* social cognitive skills in typically developing (TD) children. In Study 2, we demonstrate how this sequence can be used as a normative benchmark to investigate delay and difference in the developmental sequence of early social cognitive skills in children with neurodevelopmental disorders. We demonstrate this proof of principle using a group of autistic children (AUT), a neurodevelopmental disorder that is familiar to clinical and research communities, with a defined profile of social interaction skills and behaviours that are core to diagnosis (American Psychiatric Association 2013; World Health Organization 2018) and whom show difficulties in later developing social cognitive abilities (i.e. ToM; Baron-Cohen 1995; Baron-Cohen et al. 1985; Kimhi 2014) (Study 2).

It is well-established that some ToM tests (e.g. understanding of other’s beliefs, desires, intentions etc.) are easier than others: for example, on average, children successfully judge what others do or don’t know before they make successful judgements about others’ false beliefs (Wimmer and Perner 1983). However, such group-level patterns do not entail that individual children will reliably pass tasks in the same order. When there are good theoretical grounds for supposing that abilities in a particular domain may be developmentally related, scaling analysis makes it possible to test whether tasks in that domain are indeed passed in a stringent cumulative sequence (Guttman 1944/1950; Guttman and Greenbaum 1998). Wellman and Liu (2004) used statistical scaling to assess whether five ToM tasks passed in a reliable order within individuals aged 2 to 7 years. The outcome was reliable scaling whereby children tended to pass all tasks up to a certain point, and then failed all subsequent tasks. Wellman and Liu concluded that the cumulative structure indicated that early skills may be required for later skills to develop through a process of modification, in which early understanding broadens throughout development to encompass later understanding, or mediation, in which earlier abilities scaffold the development of later abilities (Wellman 2014; Wellman and Liu 2004; Tomasello 2014; Perner 1991; Flavell 1972).

As well as being theoretically informative, scaling tasks provides a normative benchmark and a robust tool to investigate social cognitive development in neurodevelopmental disorders. When the same set of tasks were applied to AUT children, results revealed both a delay in understanding a range of mental state constructs *and* a different scalable pattern of skill acquisition (Peterson et al. 2005). AUT children passed a task assessing their understanding of *hidden emotion* prior to passing *false belief* tasks, whilst the opposite pattern was revealed in TD children. This suggests that AUT children use different strategies in their acquisition of particular ToM skills compared to TD children (Peterson et al. 2005). These findings highlight the utility of applying scaled tasks of social cognitive skills in those with neurodevelopmental disorders by (1) identifying differences as well as delay in their social cognitive development, (2) identifying abilities that they *do* have the capacity to understand as well as those that they do not and (3) helping refine hypotheses for future research as to why social cognitive development is disrupted in AUT children.

However, whilst the earliest mentalising ability that the scale can assess typically emerges from around 2 years (Repacholi and Gopnik 1997), much evidence suggests that social cognition and perhaps even the foundations of ToM lie significantly earlier in development (Tomasello et al. 2005; Rakoczy et al. 2005). In addition, many children with neurodevelopmental disorders do not have the intellectual ability to engage in many of these tasks. Tomasello et al. (see e.g. Tomasello 2014; Wellman 2014 for recent overviews) have argued that an understanding of others’ *intentions* is a precursor to later-developing, more complex ToM constructs such as understanding others’ *belief*. This body of literature indicates that, similar to ToM understanding assessed in Wellman and Liu’s scale, distinct types of intention understanding emerge at different ages. For example, from as young as 14-months infants demonstrate understanding and motivation to assist others with their unachieved goals (Warneken and Tomasello 2006; Surin and Franchin 2017; Torréns and Kätner 2017; Rheingeld 1982; Schuhmacker et al. 2017; Dunfield et al. 2011), by 18-months infants can make inferences about the communicative intentions behind communicative cues and distinguish these from unintentional cues (Behne et al. 2005; Schulze and Tomasello 2015; Liebal et al. 2009) and by 24-months infants coordinate and cooperate with others in problem-solving activities, and thus are considered to have developed a ‘shared intentionality’. Finally, from 24 months’ infants further develop their shared intentionality to achieve mutual goals that are inherently social (i.e. to carry out a task for mutual enjoyment), as opposed to a simple mutual desire to obtain a tangible object (as in a problem-solving task) (Warneken et al. 2006; Ashley and Tomasello 1998; Brownell and Carriger 1990; Fletcher et al. 2012).

Similar to the ToM concepts studied by Wellman and Liu, these early social cognitive abilities are considered to not vary just in difficulty for infants. Instead, the abilities assessed by easier tasks (e.g., assessing basic intentions behind other’s goal directed actions) are hypothesized to be the foundations for developing a shared intentionality and cooperation skills that emerge later in development. To develop a ‘shared intentionality’, an individual must first acquire earlier developing understanding of goals behind a range of intentional actions, as well as a species unique motivation to share and represent these psychological states with another, in order to reciprocally and appropriately respond within a given scenario to achieve a joint goal (Tomasello et al. 2005; Rakoczy et al. 2005; Call and Tomasello 2008). This theoretical framework suggests that infant’s performance on these tasks may be appropriate for assessment with a cumulative scale.

To date there is no battery of tasks that assesses the developmental progression of children’s understanding of *intention* and *shared intentionality.* Whilst there are common measures that assess very early social-communicative skills, such as *The Early Social Communication Scale* (Mundy et al. 2003/2013; Seibert et al. 1982), these scales do not address the development of children’s understanding of other people’s minds specifically. Thus, it is not clear whether early social cognitive skills are acquired in a consistent developmental sequence across individuals, as observed in the more advanced ToM skills assessed by Wellman and Liu’s (2004) ToM Scale. Despite the value of Wellman and Liu’s (2004) scale, the processing demands of the individual tasks limit their ability to identify and investigate ToM abilities in younger children and children with intellectual disability. A battery of tasks assessing early social cognition would therefore complement and expand the ToM scale produced by Wellman and Liu (2004), offering opportunities to investigate difference as well as delay in neurodevelopmental disorders associated with intellectual disability whom have difficulties in social cognition and social interaction.

Study 1 reports the development of a battery of tasks to explore the developmental sequence that TD infants acquire early social cognition skills’. Study 2 is a proof of principle study to demonstrate how this battery can be applied to children with neurodevelopmental disorders, using a group of AUT children as an example. Detailing the developmental sequence of early social cognitive skills in these two groups has implications for understanding the mechanisms through which these skills are acquired; the same developmental sequence in both populations would lend weight to an argument that earlier-developing skills form the foundation for skills that are acquired later, whilst different patterns of development might suggest that these skills can develop independently. Furthermore, investigating early social cognitive development in AUT has important clinical implications, potentially highlighting potential areas of strength or difficulty to guide assessment, intervention and future research.

## Study 1

In study 1, we hypothesise that infants’ patterns of passes and fails on tasks taken from the current literature that assess different types of intentionality understanding will conform to a cumulative scale in an order of difficulty that corresponds to age approximations reported in previous literature. Specifically, we hypothesise that infants will help others obtain an out-of-reach object (observed in 14-month olds; Warneken and Tomasello 2007) and understand which object another person wants based on that person’s previous experience with an object that the person and infant were previously jointly engaged in (14-months; Moll and Tomasello 2007), before understanding another person’s implicit intentions (18-months; Bellagamba and Tomasello’s 1999) and another’s communicative intention behind a pointing gesture directing the child to the location of a hidden toy (Behne et al. 2005). These abilities will develop prior to understanding the communicative intention of another’s gaze also used to direct the child’s attention to a hidden toy (24 months; Behne et al. 2005) and demonstrating a shared intentionality to coordinate with a partner in a problem-solving task (24 months). Finally, infants will be able to coordinate with another during a social game (Warneken et al. 2006). These findings would provide stronger evidence that the social cognitive abilities assessed by these tasks may be developmentally related to one another, and that skills that develop early (e.g. basic intention reading) may form the foundations for those that develop later (e.g. cooperating with others) through a process of moderation or mediation (Hughes 2011; Tomasello et al. 2005; Rakoczy et al. 2005; Call and Tomasello 2008). They would also provide a normative benchmark of the typical sequence in which these very early developing abilities develop.

### Method

#### Participants

Participants were recruited from 13 nursery schools. Only children reported by school staff to have no developmental conditions were recruited. 98 infants were initially recruited; however, 12 infants were not tested due to an inability to settle with the experimenters. The final sample therefore consisted of 86 infants (M_age_ = 22 months, range 14–34 months; 46 male).

#### Tests of Early Social Cognition

Individual tests assessing early social cognition were selected from a well-established program of work led by Tomasello et al. (see e.g. Tomasello 2014; Wellman 2014 for recent overviews). Tests were chosen to assess different forms of explicit social cognition, hypothesised to range in difficulty across ages 1 to 3 years. In addition, to ensure practicality of administration, the tests selected required no more than two experimenters and used simple materials that could be easily constructed and transported to test sites. The selected tasks are described below (see Online Resource 1 for full administration and scoring instructions).

The *Helping* test was based on tasks developed by Warneken and Tomasello (2006, 2007). The *Helping* test contained two experimental trials, in which participants observed an experimenter make an unsuccessful attempt to reach for an object (e.g. a pen ‘accidentally’ dropped on to the floor). To pass the task, the infant was required to pass the reached-for object to the experimenter in one of the two experimental trials. Warneken and Tomasello (2006, 2007) demonstrated that children typically demonstrate such helping behaviour by 14 months. Corresponding control trials were also administered to ensure that children were correctly interpreting the experimenter’s intention to access the object in the experimental trials. In these control trials the experimenter’s behaviour was matched to that observed in the experimental trials but ‘helping’ behaviour was not required (e.g. the experimenter deliberately threw a pen on to the floor and made no attempt to reach for it).

The *Seeing-is knowing* task was based on the ‘joint attention’ condition used by Moll and Tomasello (2007), which assessed whether the infant could understand what another knows based on their previous experiences. Infants took part in two experimental trials, which used the same experimental procedure but different toys. In each trial Experimenter 1 and the infant played with a toy together for 60 s. Experimenter 2 then removed the object and placed it on a tray. This procedure was repeated with a second toy, following which Experimenter 1 announced they were leaving and left the room. Experimenter 2 and the child then played with a third (target) toy, before placing this on the tray with the previous two toys. Experimenter 1 returned to the room and exclaimed “Oh look, look at that! Wow! Look at that!”, pointing towards the tray. Experimenter 2 held the tray towards the infant and Experimenter 1 added “Wow…can you pass it to me?” with an outstretched hand. In order to pass the task, infants were required to give the experimenter the target toy in both experimental trials. Infants were expected to pass this task by 14 months of age (Moll and Tomasello 2007).

The *Re-enactment of Intended Acts* task was based on Bellagamba and Tomasello’s (1999) demonstrate intention’ conditions, which assessed the social cognitive ability to infer another’s intentions from their goal-oriented (but unsuccessful) action. The task consisted of three experimental trials. In each trial, the experimenter made three ‘unsuccessful’ attempts to perform a target act using a pair of objects. For each trial, after observing the examiner’s failed attempts, the child was given the pair of objects accompanied by the words “Oh look what I have here”, “What’s this?” or “Now it’s your turn”. Participants were required to successfully reproduce two of the three target acts in order to pass the task. Infants typically pass this task by 18 months of age (Bellagamba and Tomasello 1999).

The *Understanding Others Communicative Cues* tasks (*Communication*, Behne et al. 2005) assessed whether an infant could understand the use of pointing (*Communication: Point)* and eye gaze *(Communication: Gaze)* to direct the infant’s attention to a referent object. In both the *Communication: Point* and *Communication: Gaze* tasks, Experimenter 1 showed the child a toy, before saying “Now I’ll hide it” and placing it in one of two boxes concealed behind a movable screen. Experimenter 2 indicated to the child that she was watching Experimenter 1 hide the toy by alternating her gaze between the child and the boxes and saying “I can see”. Once the toy was hidden, the screen was removed. Whilst Experimenter 1 was turned away, Experimenter 2 provided a communicative cue (Point: extending index finger toward the correct box; Gaze: gazing between the correct box and back to the infant) to indicate the location of the toy, along with raised eyebrows to express intent. Two experimental trials were administered for each communicative cue, and participants were required to correctly identify the location of the toy in both experimental trials in order to pass the task. TD infants have been shown to pass the *Communication: Point* and *Communication: Gaze* tasks by 18 months and 24 months respectively (Behne et al. 2005). Corresponding control trials were designed to ensure that correct responses were not due to low-level attentional cues. In these control trials, after removing the screen, Experimenter 1 gave one of two non-communicative cues. This was either a ‘distracted point’ (hand held out with an extended index finger but looking down with an expression indicating preoccupation with something on the hand), or a ‘control gaze’ (gazing at the box with an absent minded facial expression). Participants were administered two control trials for each communicative cue (Point and Gaze). Whilst it could be argued that the critical difference between the experimental and control trials lay only in the number of cues available for interpretation, we followed the well-established ways that these tasks have been used and interpreted in the current literature by assuming that the crucial difference was in the way the cues had to be interpreted; only in the experimental conditions do children infer that the communicative act was intentional and relevant to the current context (Behne et al. 2005; Schulze and Tomasello 2015; Liebal et al. 2009).

The *Cooperation* tasks from Warneken et al. (2006) were used to assess whether infants could develop shared intentionality to achieve a joint goal with another individual. In the *Cooperation* tasks participants were required to: (a) cooperate with an adult in pursuit of a shared goal, and (b) evidence an attempt to re-engage their partner when the adult interrupted this joint activity. Experimenter 1 and 2 initially carried out a demonstration of the task. For *Cooperation: Tubes*, each individual pulled a handle at either end of two overlapping tubes in the opposite direction (i.e. pulling away from one another), in order to release a toy contained within the inner tube. In the *Cooperation: Trampoline* task, two individuals were required to work jointly in order to bounce an object on a hand-held trampoline. For each task the demonstration was followed by four trials. In Trial 1 and Trial 2, the child was required to engage jointly with Examiner 1 as observed. In Trial 3 and Trial 4, however, after beginning the activity, Experimenter 1 then ceased performing their role, letting go of the object and looking down with their hands on the floor. Experimenter 1 held this position for 15 s, after which they resumed their role as before. During this ‘interruption’ period, the child’s actions were coded by Experimenter 2 for attempts to re-engage Experimenter 1 in the task. A score of 0–3 was given to indicate different levels of coordination (*Tubes* task) or engagement (*Trampoline* task). In order to pass the task children were required to score a median of three for Coordination/Engagement, and to display at least one attempt at re-engagement during the interruption period (see Online Resource 1 for coding schema). Infants aged 24 months and older have been shown to achieve success on the *Cooperation* tasks (Warneken et al. 2006).

#### Procedure

Infants were tested in a quiet room in their nursery. Experimenters played with each infant for 10–15 min prior to assessment to ensure that infants felt comfortable. Tasks were administered in one of four orders, each beginning with two tasks deemed engaging (namely *Seeing-is-knowing* and the *Cooperation* tasks) to encourage infant participation and avoid early frustration. Pearson chi square tests showed no significant associations between task order and performance on any of the individual tasks (all *p* > .17). Tasks were administered over two separate test sessions to avoid fatigue.

### Results

#### Preliminary Analyses

Preliminary analyses of the *Helping*, *Communication: Point* and *Communication: Gaze* control trials were conducted to ensure that successful performance reflected an understanding of *intention,* rather than a response to low-level attentional cues (see Online Resource 2). Analysis of children’s performance during *Helping* control trials indicated that helping behaviour occurred when children understood the adult’s intention to access the object and were motivated to ‘help’ the adult. Similarly, analyses showed that non-communicative control cues in the *Communication: Point and Communication: Gaze* tasks were not sufficient to direct participants’ attention to the location of the toy, and therefore that successful task completion occurred when children understood the *intention* behind the examiner’s communicative cue during the experimental trials.

#### Task Performance

The pass rate for each task corresponded with previous literature with the exception of Seeing-is-knowing, which was expected to be similar in difficulty but in fact appeared substantially harder, with only 31 children (36% of the sample) passing the task. Therefore, the task was removed from the final scale. Table 1 shows the pass rates for the remaining tasks. McNemar’s tests, applying Yate’s correction for continuity, were used to compare performance between adjacent tasks (ranked by pass rate). Results showed that significantly more infants passed the *Helping* task than the *Communication: Point* task; the *Communication: Point* and *Re-enactment of Intended Acts* tasks were passed by significantly more infants than the *Communication: Gaze* task; and the *Gaze, Cooperation: Tubes* and *Seeing-is-knowing* tasks were significantly easier than the *Cooperation: Trampoline* task. No significant differences were found between performance on the *Communication: Point* and *Re-enactment of Intended Acts* tasks, between the *Communication: Gaze* and *Cooperation: Tubes* tasks, or between the *Cooperation: Tubes* and *Seeing-is-knowing* tasks.

**Table 1:** The percentage of infants that passed each task in the battery (excluding Seeing-is-Knowing) and the pairwise comparison results between tasks in ascending order

Task	Pass rate (%)	Number of passes	Number of fails	Pairwise comparisons	Significance level following bonferonni-holm corrections
Helping	88	76	10	*p* ≤ .001**	< .001
Communication: Point	67	58	28		
				*p* = .575	
REI	63	54	32		
				*p* = .005**	.025
Communication: Gaze	43	37	49		
				*p* = .473	
Cooperation: Tubes	37	32	54		
				*p* = .011*	.044
Cooperation: Trampoline	28	19	67		

*Indicates *p ≤* .05

**Indicates *p ≤ *.01

The order of task difficulty (according to percentage pass rate) corresponded with the expected developmental progression based on previous literature, with the exception of the *Seeing-is-knowing* task. The *Seeing-is-knowing* task was expected to be of similar difficulty to the *Helping* task, however, in the current sample the *Seeing-is-knowing* task was significantly more difficult than four other tasks. It is possible that this discrepancy was a function of the modifications made to the toys used in the *Seeing-is-knowing* task, which were chosen to be more appealing to infants than those used in the original studies. Observation during testing indicated that many infants had a strong preference for particular toys, which they subsequently selected when the experimenter requested an item. Given the discrepancy between the current results and the previous literature, and the lack of clarity regarding this discord, the *Seeing-is-knowing* task was removed from subsequent scaling analyses.

#### Guttman Scaling Analyses

The above analyses indicate a sequential task progression at a group level, but do not identify whether participants passed these tasks in reliable sequence on an individual level. Guttman scaling analyses were used to determine whether these tasks formed a reliable ‘scale’, such that success on more ‘difficult’ tasks is achieved only by children who have also passed the preceding ‘easier’ tasks. In determining whether tasks are scalable, scalogram analyses take two factors into account. The first, the co-efficient of reproducibility, assesses the extent to which the sequence of task passes/fails diverges from a ‘perfect’ scale (i.e. in a perfect scale, after failing one task a child would fail all subsequent tasks). According to Green’s (1956) method, a co-efficient of reproducibility ≥ .90 indicates a reproducible scale. The second, the index of consistency, assesses whether the co-efficient of reproducibility is above what could be expected by chance. Green suggests an index of consistency ≥ .50.

**Fig. 1:**
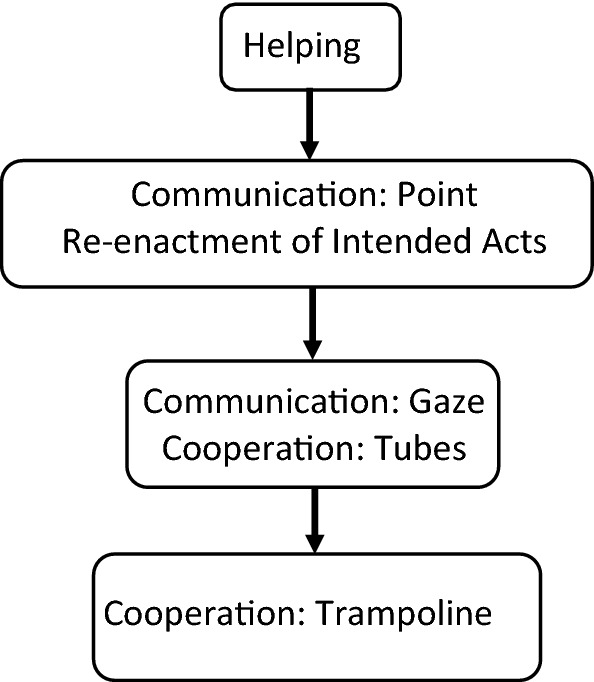
Four-stage scale of early social cognitive skills

To account for tasks of similar difficulty (i.e. tasks for which the pass rate was not significantly different), a pass was assigned if the infant passed *either* of the two tasks (i.e. if they passed *Commuication: Point* OR *Re-enactment of Intended acts;* or if they passed *Communication: Gaze* OR *Cooperation: Tubes*). This produced a four-stage scale (see Fig. 1).

For this four-stage scale, 88% of participants demonstrated a pattern of performance corresponding to the scale exactly (see Table 2). The co-efficient of reproducibility was 0.96, with an index of consistency of .5, indicating a reliable scale.

**Table 2:** Guttman scalogram patterns

Task	Pattern					
	0	1	2	3	4	Other patterns
Helping	–	+	+	+	+	
REI or communication: Point	–	–	+	+	+	
Communication: Gaze or Cooperation: Tubes	–	–	–	+	+	
Cooperation: trampoline	–	–	–	–	+	
Number of cases	4	7	19	29	14	13
Average age (m)	14.8	16.9	19.7	23.6	27.1	21.5
Age range	14–17	14–23	14–27	15–33	22–34	14–27

## Summary

Study 1 used tasks designed to assess an understanding of *intention* and *shared intentionality* to explore the acquisition of early social cognition in infants. These tasks were chosen as they assess skills that have been hypothesised to show cumulative development, and to provide foundations for later-emerging social abilities (Tomasello et al. 2005; Rakoczy et al. 2005). Six of the seven tasks selected showed levels of performance consistent with previous reports in the literature. Analysis of a four-stage sequence accounting for tasks of similar difficulty showed that infants passed these tasks in a reliable and scalable fashion, thus forming an Early Social Cognition Scale (*ESCogS*).

## Study 2

The aim of Study 2 was an exploratory study to demonstrate proof of principle of how the *ESCogS* can be used as a normative benchmark to investigate whether AUT children show a typical or atypical sequence of development on this task. An important feature of using a scale in this way is that no matched control group is necessary because conclusions are based upon whether the pattern of performance within the AUT group is the same as the normative pattern, irrespective of the absolute levels of performance. Existing studies suggest that AUT children perform as well as control groups on some tasks of early social cognitive skills such as those requiring re-enactment of intended (but unsuccessful) acts (Aldridge et al. 2000; Carpenter et al. 2001), and those requiring demonstration of helping behaviour (Liebal et al. 2008). However, AUT children have been reported to demonstrate difficulty with other tasks that required a shared intentionality such as coordination of actions with an adult in cooperative problem-solving games (Liebel et al. 2008), and those requiring joint attention (Baron-Cohen 1995; Leekam et al. 1997). A lack of joint attention skills may point to difficulty interpreting the communicative intentions from others’ point and eye gaze. On the basis of this existing research, it was hypothesised that AUT children would show a different pattern in the acquisition of early social cognitive skills compared to TD infants, with some skills apparently intact, such as instrumental helping, whilst others are absent or significantly delayed.

### Method

#### Participants

Children with a diagnosis of ASD were recruited primarily through a special educational needs schools. The parents/carers of 23 children consented to participation in the study. Six additional participants were recruited from a participant database held by the Cerebra Centre for Neurodevelopmental disorders at the University of Birmingham. These participants were contacted initially to participate in other studies at x, and once enrolled were given the opportunity to take part in this study.

Wellman and Liu's (2004) ToM Scale was initially administered to eight participants, out of the total 29, who were reported to be verbally fluent by school staff. These data are not included here as the present study focuses on data from the *ESCogS*. If children failed two of the first three tasks of Wellman and Liu's (2004) battery (*diverse desire*, *diverse belief* or *knowledge access)*, they were subsequently assessed using the *ESCogS*, and their data were included in the current study (*n* = 3).

The *ESCogS* was thus administered to 24 participants. Three participants were excluded because they did not engage with the study tasks. The final sample therefore consisted of 21 children (see Table 3 for demographic characteristics).

**Table 3:** Participant data: chronological age, mental age and developmental quotients

	Mean	Range
Age (months) of TD children from Study 1	22	14–34
Age (months)	101.52	39.96–171.96
Non-verbal mental age equivalent (months)^a^	40.34	6.50–114.00
DQ based on non-verbal mental age^a^	42.86	
Verbal mental age equivalent (months)^a^	32.84	4.00–92.00
DQ based on verbal mental age^a^	37.04	4.36–106.45

^a^Mental age and DQ not available for two participants

#### Tasks and Measures

The Autism Diagnostic Observation Schedule (ADOS; Lord et al. 1989, 2012) was administered by a trained researcher. Due to the difficulty of recruiting AUT children and limited resources for the project, data for this study was collected in tandem with other studies recruiting the same group and using the ADOS. These studies used different editions of the ADOS. Therefore the ADOS was administered to five participants and the ADOS-2 administered to the remaining participants. As scores between these two versions have not been yet made comparable, scores were only used to confirm diagnosis. ADOS scores unavailable for one participant.

Mental age was assessed using either the Mullen Scales of Early Learning (MSEL) (Mullen 1995) or the British Ability Scales Second Edition (BAS-II) (Elliott et al. 1996). The most suitable assessment was selected by researchers based on the child’s chronological age, preliminary information from the school regarding the child’s verbal fluency, and perceived receptive and expressive language ability during initial contact with the child. From the MSEL participants were administered the Fine Motor and Visual Reception subscales (non-verbal), and the Receptive Language and Expressive Language subscales (verbal). From the BAS-II participants were administered the Matrices and Quantitative Reasoning subscales (non-verbal) and the Word Definitions and Verbal Similarities subscales (verbal). The MSEL (Mullen 1995) provides normative data for children with a chronological age of ≤ 66 months only, therefore age equivalent scores were used in the present study. For each participant, mean verbal and non-verbal age equivalents used in analyses were calculated by averaging scores from the two relevant subscales of the developmental assessment the participant took part in (see Table 3).

Participants were administered the six-task ESCogS developed in Study 1. Individual tasks were administered and scored as described in Study 1 (see Online Resource 1 for further detail).

#### Procedure

Participants were tested individually in a quiet room at their school. Participants that were recruited through the Cerebra Centre for Neurodevelopmental Disorders participant database were tested individually in a quiet room at the University of Birmingham. All assessments were completed by researchers trained in administration and scoring.

## Results

### Preliminary Analyses

As described in Study 1, preliminary analyses of the *Helping, Communication: Point* and *Communication: Gaze* control trials were conducted to ensure that successful performance reflected an understanding of *intention,* rather than a response to low-level attentional cues. Again, analyses indicated that the helping behaviour observed during *Helping* experimental trials reflected an understanding of the adult’s *intention* to access the object. Furthermore, control cues during the *Communication* tasks were not sufficient to direct participants’ attention to the location of the toy, indicating that successful task completion occurred when children understood the *intention* behind the experimenter’s communicative cue during the experimental trials.

### Task Performance

Table 4 presents the pass rate for each task in the *ESCogS*, alongside pass rates from the TD children assessed in Study 1. These data indicate that, at a group level, the performance of children with ASD followed a similar sequence to that observed in TD infants. The pass rate for the *Communication: Gaze* task (19.05%) *Cooperation: Tubes* (9.52%), *Cooperation: Trampoline (0%)* was much lower than the *Communication: Point* and *Re-enactment of Intended Acts* tasks (both 61.90%) in the current sample, suggesting children with ASD had particular difficulty interpreting intention in eye gaze and cooperating with the experimenter to achieve joint goals.

**Table 4:** *ESCogS* task pass rates in descending order

Task	Number of children who passed (%)	
	Aut children (N = 21) (current sample)	Typically developing children (N = 86) (from Study 1)
Helping	16 (76%)	76 (88%)
Communication: Point	13 (62%)	58 (67%)
Re-enactment of intended acts	13 (62%)	54 (63%)
Communication: Gaze	4 (19%)	37 (43%)
Cooperation: Tubes	2 (10%)	32 (37%)
Cooperation: Trampoline	0 (0%)	19 (22%)

Table 3 presents chronological age and mental age, and developmental quotients (DQ) for the current sample alongside chronological age data from Study 1. Mental age data were used in the current study to provide a more accurate measure of ability level in children with intellectual disability. The data in Table 3 demonstrate that mean mental age in the current sample was higher than the chronological age (and therefore assumed mental age) of the typically developing sample in Study 1. As such, lower pass rates in the three most difficult tasks (*Gesutres: Gaze* and both *Cooperation* tasks) in the current sample are consistent with delay or deficit in early social cognitive skills in ASD.

### Guttman Scaling Analyses

To test whether children with ASD passed these tasks in a reliable sequence corresponding to that seen in TD children, Guttman scaling analyses were performed ranking tasks according to the *ESCogS* from Study 1 (see Fig. 1). In Study 1, two pairs of tasks were considered to be of equal difficulty (*Re-enactment of Intended Acts* and *Communication: Point*; and *Communication: Gaze* and *Cooperation: Tubes*). For these task pairs, a pass was assigned if the child was successful on *either* task.

In the current sample, 90.48% of participants demonstrated a pattern of performance corresponding to the *ESCogS* precisely (see Table 5). The co-efficient of reproducibility was 0.98, with an index of consistency of .58, indicating a reliable scale. Thirteen (61.90%) participants demonstrated a single pattern of performance, in which they were successful on the first two steps of the scale (Step 1: *Helping*; Step 2: *Re-enactment of intended acts* OR *Communication: Point*) but failed all subsequent tasks.

**Table 5:** AUT group scaling and participant characteristics according to the *ESCogS* compared to chronological ages of TD children (study 1) within each pattern

Task	Pattern					
	0	1	2	3	4	Other patterns
Helping	−	+	+	+	+	
REI or communication: Point	−	−	+	+	+	
Communication: Gaze or cooperation: Tubes	−	−	−	+	+	
Cooperation: Trampoline	−	−	−	−	+	
Number of cases	3	1	13	2	0	2
Age (months) of TD children from study 1	14.8 (14–17)	16.9 (14–23)	19.7 (14–27)	23.6 (15–33)	27.1 (22–34)	21.5 (14–27)
AUT children age (months)	77 (60–109)	125 (125)	115.4 (74–172)	96.5 (82–111)	NA	40.4 (40–41)
NVMA (mean/range)	29.0 (6.5–68.5)	13.5 (13.5)	40.5^a^ (17.5–62.5)	72.8 (31.5–114.0)	NA	34.5^a^ (34.5)
VMA (mean/range)	43.3 (4.0–66.0)	7.5 (7.5)	31.04 (7.0–72.5)	131 (39.0–92.0)	NA	37.0 (37.0)

^a^Mental age data not available for one participant

To investigate the role of factors that may influence participants scale performance, participants were assigned a scale position score identifying the scale step of the hardest task they had passed. Developmental quotients (DQ) based on the ratio between children’s chronological age and their verbal and non-verbal mental ages were calculated. Kendal-tau correlation analyses revealed that both verbal (τ_b_ (18) = .388, *p* = .037) and non-verbal DQ (τ_b_ (18) = .461, *p* = .013) had a moderate positive association with participant’s scale position score. To investigate whether these associations were driven primarily by chronological or mental age, correlations were run between verbal mental, non-verbal mental and chronological age, and scale position score. Whereas verbal mental age showed a moderate positive correlation with scale position score (τ_b_ (18) = .385, *p* = .041), chronological and non-verbal mental age did not.

## Summary

In summary, analysis of performance on *ESCogS* revealed a reliable Guttman scale in children with ASD, paralleling the performance of TD children observed in Study 1. Correlational analyses showed that overall performance on the *ESCogS* was positively associated with verbal and non-verbal developmental quotients. However, verbal mental age but not non-verbal or chronological age were correlated with scale position score.

## Discussion

Study 1 used tasks from an established body of research assessing an understanding of *intentions* and *shared intentionality* to explore the development of early social cognitive skills in infants. Using Guttman scaling, this is the first study to show that these skills develop in a consistent developmental sequence, thus forming a reliable Early Social Cognition Scale. The sequence showed that children first display helping behaviour, followed by the ability to understand a pointing gesture and an understanding of another’s implicit intentions and goals. These skills precede the ability to understand the communicative content of gaze, and to co-ordinate with another person in a problem-solving task, and finally the ability to cooperate with another person during a social game.

Reliable sequences may indicate a process of modification, in which earlier-developing skills are generalized to include those developing later, or a process of mediation, through which ‘easier’ skills form a scaffold for later developing abilities (Wellman and Liu 2004). The *EScogS* expands the Theory of Mind Scale produced by Wellman and Liu (2004), which assesses more advanced social cognitive skills. Using tasks designed to be engaging and appropriate for infants, the *ESCogS* developed in Study 1 offers opportunities for a more comprehensive perspective of social cognitive development than can be achieved through the Theory of Mind scale alone.

Study 2 applied the *ESCogS* to examine the development of early social cognitive skills in children with neurodevelopmental disorders, using a group of AUT children to show this proof of principle. Guttman scaling analyses revealed that, when tasks were ranked according to the *ESCogS*, AUT children demonstrated a reliable developmental progression consistent with that observed in typical development. The consistency between the sequences observed in TD children and AUT children lends further weight to the argument that earlier skills act as a foundation for later emerging skills in both groups.

Although AUT children produced a reliable sequence paralleling that reported in TD infants, pass rates were numerically lower on the three most difficult tasks (*Communication: Gaze, Cooperation: Tubes* and *Cooperation: Trampoline*). 61.90% of the sample displayed a single pattern of performance, in which they were successful on the first two steps of the sequence, but then failed *Communication: Gaze* (which requires following another’s eye gaze) and both subsequent Cooperation tasks (which both requires the child to look at the experimenter when attempting to re-engage them; see Online Resource 1). Given that AUT children had a higher mean mental age, this lower pass rates are clearly consistent with a delay in the development of these early social cognitive skills. Importantly, irrespective of their absolute level of performance the scaling analysis provided evidence that AUT children passed the tasks in the same developmental sequence.

It has been suggested that AUT children who pass false-belief tasks use different strategies from TD individuals (e.g., Tager-Flusberg 2016). Conceivably, AUT children who passed tasks assessing understanding other’s intentions may have done so using alternative strategies to TD infants. Whilst nothing in the current dataset can distinguish the strategies children may have used, we can evaluate the likelihood that alternative strategies would have yielded the same order of task difficulty observed in the scale by chance alone. Taking account of pairs of tasks of similar difficulty, there are 180 alternative task sequences that might have been observed, and so the chances of observing the same task order in AUT children by chance is 1/180. We therefore think it is most plausible that the observed order of task difficulty in the AUT sample arises from psychological processes that are the same or relevantly similar to those that determine the order of task difficulty for TD children.

Four out of the final six tasks (both *Communication* and both *Cooperation* tasks) included in the battery required gaze following. AUT children show atypical gaze following (Lynch et al. 2013), and reduced eye contact with others is a characteristic feature of AUT and is recognised within ‘gold standard’ diagnostic instruments such as the ADOS (Lord et al. 2012). AUT children may have subsequently been disadvantaged on these tasks due to poor gaze following rather than difficulties in intention reading per se. However, gaze following is a basic skill that ‘transforms’ into joint attention to become a fundamental component for shared intentionality by enabling sharing of common ground and mutual knowledge with another person in order to facilitate cooperation (Tomasello et al. 2005; Tomasello and Carpenter 2007; Tomasello and Carpenter 2007). It is then perhaps unsurprising that many of the tasks in the *ESCogS* require some form of gaze following and fits into the theoretical framework of the development of shared intentionality.

The sequence step that children reached was found to be positively associated with both their verbal and non-verbal DQs. However, only verbal and not non-verbal mental or chronological age was found to be associated with children’s sequence point position. These findings are interesting considering that tasks were chosen and designed to have minimal language demands. However, social cognition and intention reading has been hypothesised to be vital for word learning and language acquisition (Tomasello 1992, 2000, 2003; Brooks and Meltzoff 2008). Previous studies indicate that performance on intention reading tasks are associated with receptive and expressive language (Peters-Scheffer et al. 2018) and the ability to follow another’s gaze predicts accelerated vocabulary growth in the first 2 years (Brooks and Meltzoff 2008). Subsequently, the discrepancy observed in many children with autism in which verbal ability is poorer relative to non-verbal ability (Mayes and Calhoun 2016; Ankenman et al. 2014; Barbaro and Dissanayake 2012; Nowell et al. 2015) may be influenced by poor social cognition. Future work should aim to replicate these findings in a larger sample of AUT children and elucidate the factors that may contribute to individual differences that influence children’s sequence progression, including age and ability as well as AUT specific characteristics such as symptom severity and specific skills such as joint attention.

We investigated ToM-precursors (i.e. intentionality abilities) based on literature illustrating the association between ToM abilities and social behaviour (Kimhi 2014). However, we acknowledge that this is not the only theoretical approach to studying typical and atypical social and communication behaviours. Approaches that emphasise variation in social motivation (Chevallier et al. 2012), traits for empathizing-systemising (E-S) (Baron-Cohen 2009), and executive capacity (Russell 1997), emphasise different distal causes for variation in ToM abilities, but are essentially compatible with a ToM approach. In contrast, “embodiment approaches” explicitly reject the ToM approach. Instead they suggest that social communication difficulties are primarily due to sensory-motor problems, which lead to reduced sensorimotor input and feedback during socially interactive contexts. Subsequently, the individual does not encode a strong neural representation of these contexts and their responses are less automatic and efficient within social situations (Eigsti 2013; De Jaegher 2013; Gallagher and Varga 2015). The current work was not designed to distinguish the ToM approach from approaches that take an embodied perspective, and we acknowledge that our conclusions would not follow if one were to reject the ToM approach entirely.

The above discussion should be considered in light of a number of theoretical and practical considerations. Firstly, it is important to note that a reliable Guttman scale does not, in itself, denote the progressive development of a single underlying trait or concept. Indeed, a Guttman scale can, theoretically, be produced by measuring performance across isolated tasks, if these tasks are sufficiently varied in difficulty. Alternatively, a reliable Guttman scale in the current study could represent the development of another, unmeasured skill, such as working memory or executive functioning (Wellman et al. 2011). Nonetheless, the study provides important new insights into the *sequencing* of these early social cognitive skills in children with ASD, which cannot be obtained from studies that solely compare group means on individual tasks.

This is the first study to demonstrate that early social cognition tasks are passed by TD infants and AUT children in a scalable progression. However, due to limited project resources, sample size of the AUT group was small, meaning that it will be important for future work to determine whether the reliable developmental sequence observed generalises to the broader population of AUT children. The lack of a comparison group of children with intellectual disability *without* AUT meant that we could not test whether the AUT children were impaired compared with a relevant comparison group. Participants in Study 1 and Study 2 were not matched to one another. The range of ages assessed by the *ESCogS* assesses abilities that typically emerge over the first 3 years of life. However, most AUT children in the UK do not receive a diagnosis until they are about 4.5 years old (Brett et al. 2016). In order to recruit individuals with a diagnosis of ASD, this necessarily resulted in a sample of children of an older chronological age with some level of intellectual disability, which introduced other group-level differences. However, to investigate whether the *developmental sequence* of social cognitive abilities scale *within* a cohort, scaling analysis requires only that participants included have a wide range of mental age that at least span the ages that TD children pass these tasks (Guttman 1950). Despite the wide range of age and differences in chronological and mental age between TD infants and AUT children, the sequence in which children passed these tasks still followed the same stringent cumulative sequence observed in typically development as assessed by Guttman scaling. These findings provide further support that this sequence is robust, regardless of the disparities between individuals age and mental age in the AUT group. Importantly this demonstrates the utility of the scaling approach for evaluating social cognition in groups that may vary widely in age and ability and whom may be difficult to match on all relevant parameters.

Despite the limitations above, this study offers significant new contributions to the literature. Findings demonstrated that early social cognitive skills develop in a consistent trajectory in TD infants and preliminary evidence AUT children pass these tasks in the same sequence, indicating that earlier-developing skills provide a foundation for those acquired later in development. This is the first study to explore the developmental progression of early social cognitive skills, compared to previous research focusing on the development of individual skills at a group level. Furthermore, this novel study explores how the *ESCogS* can be used to explore the developmental progression of these early skills in clinical groups at high risk of impairments in social cognition, such as AUT children, considering how this compares to a TD normative sample. Given the links between these early skills and later, more-complex mentalising abilities, understanding the development of early social cognition has a key role to play in delineating ToM development and its impact on social interaction and communication.

## Electronic supplementary material

Below is the link to the electronic supplementary material.

Supplementary file1 (DOCX 27 kb)

Supplementary file2 (DOCX 13 kb)
