# Supplementary material for: Scaling of Early Social Cognitive Skills in Typically Developing Infants and Children with Autism Spectrum Disorder
Source: J Autism Dev Disord. 2020 Mar 18;50(11):3988–4000. doi: 10.1007/s10803-020-04449-9 (PMC7557487; doi:10.1007/s10803-020-04449-9)
Supplement: Supplementary file 1 — Supplementary file1 (DOCX 27 kb) [file 10803_2020_4449_MOESM1_ESM.docx]

**Online Resource 1**

**Tests of early social cognition: Administration and scoring**

**Tasks, materials, and scoring**

We selected a range of tasks from a well-established program of work led by Tomasello, Carpenter and colleagues for the following reasons. Success or failure in these tasks represent the presence or absence of a more basic set of social cognitive abilities that develop prior to traditional ToM competencies. Specifically, these tasks aim to assess types of intention understanding, which requires an individual to understand another person’s goal and their action plan to achieve that goal (Tomasello et al., 2005). In addition, in common with other such task batteries (e.g. the Mullen Scales of Early Learning) (Mullen, 1995) these tasks have the useful property of being based upon simple materials that can be transported and require no more than two experimenters.

Tasks were selected from a review of the literature of tasks assessing early explicit social cognitive skills according to two principal criteria: 1) they should assess putatively different components of early understanding of intentionality, and 2) they should range in difficulty across ages from one to three years. In addition, for practical reasons we selected tasks that used simple equipment and required no more than two experimenters, so that the tasks could be administered easily in a nursery or (for future work) in participants’ homes. Table 1 outlines the tasks and ages of success observed in the original studies. Although these studies did not identify precise ages that abilities appear or whether these ages generalised over different populations, these age approximations indicated which tasks were likely to be passed earlier or later. Task methods were based closely upon the original studies, with minor modifications to fit a within- rather than between-subjects design. While all tasks had either multiple trials or multiple scoring criteria, scaling analysis requires each child to be assigned a pass or fail for each task. We rejected a liberal passing criterion, in which one instance of the target behaviour was sufficient for passing, because the 50% guessing rate on two tasks (Gaze and Point) greatly inflated the pass rate, so that performance on these tasks appeared much better than in the reported literature. We rejected a stringent passing criterion, in which at least two instances of every target behaviour were necessary for passing, because some behaviours (such as helping) were unlikely to occur by chance, and the strict criterion risked underestimating performance. Our final scoring criteria, described below, struck a balance between these considerations. Importantly, however, the serial order of task difficulty was the same under all scoring schemes, with the exception of *‘gaze’* and *‘point’* under the liberal passing criterion, where the inflation due to guessing resulted in these tasks being passed at the same rate as *‘helping’*.

**Table 1. Predicted age of acquisition for each task**

| Task name | Age of acquisition (in months) |
| --- | --- |
| Helping | 14 |
| Seeing-is-Knowing | 14 |
| Re-enactment of Intended Acts | 18 |
| Communication - Point | 18 |
| Communication - Gaze | 24 |
| Cooperation - Tubes-with-handles | 24 |
| Cooperation - Trampoline | >24 |

**Helping**

The *‘out of reach’* helping tasks designed by Warneken & Tomasello (2006; 2007) determined whether infants possessed the social cognitive ability to understand another person’s intentions and unachieved goals, and their altruistic motivation to act on behalf of another.

*Control trials*

Following a short warm up period infants were sat at a table opposite Experimenter one (E1). For the ‘pen’ condition, E1 was seen to use a pen for drawing. The experimenter then stopped drawing, put the lid on the pen, intentionally threw it on the floor and did not reach for it. For the ‘paper balls’ condition, E1 set up three paper balls on her side of the table and three paper balls on the infant’s side of the table. The experimenter then sat back down and picked up each of the paper balls ‘one-by-one’ using a pair of tongs and then placed them back on the table.

*Experimental trials*

For the ‘pen’ condition, Experimenter two (E2) was seen to use a pen for drawing, she then ‘accidentally’ dropped the pen on the floor and unsuccessfully reached for it. For the ‘paper balls’ condition, E2 picked up each paper ball with a pair of tongs and placed them in a cardboard container. She then attempted to reach for the three paper balls on the infant’s side of the table but failed because they were too far away.

*Coding*

In each trial, infants’ behaviour was coded for whether or not they performed the required *target* behaviour. For the pen trial this involved the infant passing the pen back to the experimenter. For the paper balls trial, it involved the infant passing or pushing the paper balls towards the experimenter.

*Pass/fail criterion and rationale*

Infants were coded as having ‘passed’ the helping task if they successfully demonstrated one of the target behaviours. This was considered appropriate as unlike some of the other tasks included in the scale, ‘helping’ behaviour was considered unlikely to occur by chance. Therefore, one demonstration was deemed sufficient to indicate that the infant had acquired the skill and passed the task.

**Seeing-is-knowing**

The ‘joint attention’ condition used by Moll & Tomasello (2007) assessed whether an infant could understand that an individual would know about an object that the infant and the individual were previously jointly engaged with. For this task each infant first received a pre-test to ensure that they could understand what was to be asked of them in the subsequent experimental trials. The infants then received two experimental trials, which were administered over two separate testing sessions. Each trial involved the same experimental procedure but different toys were used.

*Pre-test*

In the pre-test the infant sat at a table with two experimenters and played with a ball, a teddy bear and a toy car one at a time for 30 seconds each. E2 then placed each toy on a tray and held it out in front of the infant. E1 then requested the infant pass each toy successively by name. To pass the pre-test the infant had to successfully pass at least one of the first two toys requested by the experimenter.

*Experimental trials*

In each trial the infant sat a table with two experimenters. E2 began the trial by bringing out the first toy, handing it to E1, and saying “Look what I’ve got here”. E1 and the infant then played with the toy together for 60 seconds. During this time E1 showed the infant that the toy could make an interesting sound if you handled it a particular way. The experimenter also made comments during the paly such as “Oh look at that”, “That’s exciting” and “How nice!”. Following the 60 seconds play, E2 took the object and placed it on a tray saying “I’ll put this here now”. E2 then brought out a second toy and the procedure was repeated. After play with the second toy had finished and E2 had placed it on to the tray, E1 announced that she was leaving, saying “I am going outside now – bye bye”, and left the room. After E1 had left, E2 then looked at the infant and said “E1 is outside, she can’t see us, but we’ll keep playing anyway”. E2 then brought out the third and final (target) toy and played with the infant for 60 seconds. After 60 seconds E2 placed the toy on the tray along with the other two toys saying “I’ll out this here now”. At this point E1 returned to the room and exclaimed “Oh look, look at that! Wow! Look at that!”, pointing in the general direction of the tray, which E2 then held towards the infant. E1 then added “Wow…can you pass it to me?”, with an outstretched hand pointed in the general direction of the tray. E1 repeated this request up to five times if necessary. During this time E1 did not look directly at the target object but looked at the infant or the general direction of the tray.

*Materials*

The materials in each trial consisted of toys that were easily distinguishable by colour and shape. All toys were of similar size and made a sound or action when manipulated in a particular way. In trial one the toys used were: a tambourine with a mirror on the back, a maraca, and a yellow block with a button on the top which opened a door on the side of the block when pressed (target toy). For trial two the toys used were: a soft snail rattle with a stretchy tail and ears, a water filled jelly car with sparkles that moved when shaken, and a blue block with a button on the top that made a butterfly and bee spin round when pressed (target toy).

*Coding*

For each trial the infant’s behaviour was coded for whether or not they passed the target item to the experimenter.

*Pass/fail criterion*

Infants were coded as having passed the task if they successfully passed the correct target item to the experimenter in *both* experimental trials. This pass/fail criterion was deemed necessary to reduce the possibility that infants might ‘pass’ the task by selecting the correct item by chance. It was considered far less likely that infants would select the correct item by chance on *two* experimental trials as the chance of this happening is below 50%.

**Re-enactment of intended acts**

Bellagamba and Tomasello’s [20] *‘demonstrate intention’* conditions assessed the social cognitive ability to infer other’s intentions by interpreting that person’s goal-oriented (but unsuccessful) action. For this task infants were sat at a table opposite the experimenter. Three experimental trials were administered to each infant and all followed the same procedure. For each trial the experimenter presented the infant with an object pair that could be used to perform a target act – a loop that could be hung over a protruding peg, some beads that could be dropped into a cup, or a square with a hole in it that could be stacked upon a protruding peg. For each trial the experimenter modelled the intention to perform the target act but ultimately failed to perform the act.

For the loop and peg, the experimenter picked up the loop and moved her hand towards the peg but released it inappropriately each time so that instead of hanging over the peg, the loop ‘accidentally’ fell to the table. Initially the loop was released slightly too far to the left, then too far to the right and then too low.

For the beads and cup, the experimenter picked up the beads and attempted to drop them into the cup but released them inappropriately each time so that they ‘accidentally’ fell to the table instead. Initially the beads were lowered just so that they touched the lip of the cup but then released so that they fell to the side. On the next attempt the beads were suspended too far in front of the cup and so fell to the table when released. On the final attempt the experimenter gathered the beads loosely in her hand but then scraped her hand over the top of the cup so that the beads fell to the side rather than inside the cup.

For the square and protruding peg the experimenter picked up the square and attempted to place it upon the peg, however each time the experimenter failed to align it correctly so that it ‘accidentally’ overshot the peg. Initially the square overshot to the right, then to the left and finally to the front.

After the experimenter had demonstrated the three failed attempts she offered the object pair to the infant. During the experimental procedure the experimenter did not provide the infant with any prompts or cues, however, the experimenter gained the infant’s attention by saying “Oh, look what I have here”, “What’s this?”, and “Now it’s your turn”.

*Coding*

For each trial infants’ behaviour was coded for whether or not they went on to perform the target act themselves: for the loop and peg this involved them hanging the loop over the protruding peg; for the beads and cup this involved them dropping the beads inside the cup; and for the square and peg this involved them stacking the square over the protruding peg.

*Pass/fail criterion*

Infants were coded as having passed the task if they successfully performed the target act in *two or more of the three trials.* This pass/fail criterion was deemed necessary to reduce the possibility that infants might ‘pass’ one trial simply by chance or because the apparatus ‘afforded’ a particular response from that infant. It was decided that two or more target acts were less likely to occur ‘just by chance’ and therefore it was deemed that this provided sufficient evidence that the infant possessed the social cognitive skill.

**Understanding other’s communicative cues: ‘Point’ and ‘Gaze’**

The *‘Communication: Point’* and *‘Communication: Gaze’*  tasks (Behne, Carpenter & Tomasello, 2005) assessed whether an infant could follow communicative cues to a referent object and understand that the cue was directed towards them and relevant to their current social context. Due to the identical experimental procedures used to assess these communicative cues, trials for both cues were administered together in one procedure.

*Warm up phase*

Before the task began each infant took part in a warm up phase. This was to familiarise them with the hiding procedure and the containers used. Infants were sat at a table next to E2 and across a table from E1. E1 placed a pair of open containers in front of the infant and then brought out a small toy. E1 then announced “Look, I’ll hide it”. As the infant watched, E1 placed the toy in one of the containers and then placed the lids on both. E2 then encouraged the infant to retrieve the toy by saying “Where’s the toy” and “Can you get the toy?” This warm up hiding procedure was repeated three times with three different sets of containers.

*Control trials*

Each infant participated in four control trials. These control trials were administered to check that search performance was indicative of understanding the experimenter’s intentions and not simply due to low level attentional cueing. For each control trial E1 placed a pair of open containers on the table then produced a small toy. If the infant showed interest in the toy E1 then placed a movable screen in front of the two containers, lowered the toy behind the screen and said “Now I’ll hide it”. At this point E1 then quickly pushed the containers together, hid the toy in one, and then moved them apart again. The distance between each container ensured that the infant could not grab both containers at the same time. Following the hiding procedure E1 removed the screen and gave one of two non-communicative control cues:

- *Control Point* – E1 performed a ‘distracted point’ by holding out her hand and slightly extending her index finger. E1 simply looked down at her hand with an expression that indicated she was preoccupied by something on her hand.
- *Control gaze* – E1 gazed at the container with an absent-minded facial expression, eyes unfocused with a neutral facial expression.

Following each non communicative cue E2 then encouraged the infant to retrieve the toy by saying “Where’s the toy?” and “Can you get the toy?” Each infant received two control gaze trails and two control point trials, which were represented in one of four different counterbalanced orders.

*Experimental trials*

As before, for each trial E1 placed a pair of containers in front of the infant and produced a toy. If the infant showed interest in the toy E1 then placed a moveable screen in front of the two containers, lowered the toy behind the screen and said, “Now I’ll hide it”. During this hiding procedure E2 showed the infant that she was watching by alternating her gaze between the containers and the infant then announcing, “I can see”. After hiding had been completed E1 pushed the containers apart and removed the screen. E1 then turned away from the table in order to place the screen behind her. At this point, while E1 was not looking, E2 established eye contact with the infant and gave one of two communicative cues:

- *Point* – E2 extended her index finger and pointed at the container expressing intent through raised eyebrows.
- *Ostensive gaze* – E2 gazed at the target container and then back to the infant expressing intent through raised eyebrows.

Following each communicative cue E1 then turned back to the table and encouraged the infant to retrieve the toy by saying “Where’s the toy” and “Can you get the toy?”. Each infant received two gaze trials and two point trials which were presented in one of four different counterbalanced orders.

To minimise the possibility of perseveration errors being made, each pair of containers were different in colour and shape and the same pair were never used on successive trials. For each trial, it the infant attempted to open a container but could not quite manage to, then one of the experimenters assisted the infant. Furthermore, if the infant chose the incorrect box and did not find the toy, then the experimenters opened the correct box and gave the toy to the infant. This was done to ensure that the infant did not become frustrated and disengage from the task.

*Coding*

For each trial the box that the infant first selected and attempted to open was recorded. If the infant selected the container that the toy was hidden in this was coded as correct. If the infant selected the container without the toy this was coded as incorrect.

*Pass/fail criterion*

Infants were coded as having passed the point trials if they successfully chose both of the correct containers following each point cue. Similarly, infants were coded as having passed the gaze trials if they successfully chose both the correct containers following each gaze cue. This pass/fail criterion was deemed necessary to reduce the possibility that infants might ‘pass’ the task simply be selecting the correct location by chance. It was considered much less likely that infants would select the correct location by chance on *two* consecutive experimental trials.

**Cooperation (Tubes and Trampoline)**

A cooperative problem-solving task (*‘tube-with-handles’*) and a social game (*‘trampoline’*) [17] assessed whether an infant could develop ‘shared intentionality’ to produce and achieve a joint goal with another individual. In particular, these tasks assessed 1) the skill that infants could coordinate with a partner and 2) whether infants communicated to reengage a partner who disrupted achieving a joint goal.

*Cooperation: Tubes*

In the *Cooperation: Tubes* task the infant’s goal was to retrieve a toy that had been hidden inside a tube. This tube could be pulled apart by pulling the handles at each end of the tube. However, the length of the tube made it impossible for the infant to perform this goal alone and therefore to be successful the infant was required to ‘work together’ and cooperate with the experimenter. For each infant, the experimental procedure included a number of steps.

- *Familiarisation and demonstration*: The task began with a brief familiarisation period in which the infant was shown the tube and encouraged to hold each of the handles. After the infant was familiar with the apparatus, E1 and E2 pulled the tube apart and E1 placed an attractive object inside. The two experimenters pushed the tube back together and placed it on the floor. E1 and E2 then proceeded to demonstrate how the toy could be retrieved by each of them pulling the handles at each end. Following this demonstration E1 produced another attractive object and placed it inside the tube as before, then pushed it back together with E2.
- *Experimental trial one*: Following the familiarisation and demonstration period E1 invited the infant’s participation by alternating gaze between the infant and the tube. If the infant was immediately successful and cooperated with the experimenter to open the tube then trial 2 was administered. However, if the infant was not successful within 30 seconds E1 and E2 carried out the demonstration phase again. Following the second demonstration the infant was encouraged to participate again, this time using verbal cues such as “Come and try” and “Look, tube!”. If the infant was still unsuccessful the demonstration was repeated for a third time but this time E2 also encouraged the infant to stand by her and hold the handle together with her. If the infant was unsuccessful after three demonstrations the task was discontinued.
- *Experimental trial two*: Following trial one, E1 produced another toy and placed it inside the tubes as before. The infant’s participation was then prompted by E1. Once object was retrieved trial three was administered. If the infant was not successful after 60 seconds then the task was discontinued.
- *Experimental trial three*: Following trial two, E1 produced another toy and placed it inside the tube, as before. The infant’s participation was once again prompted by E1. However, in this trial, unlike trials one and two, when the infant picked up their side of the tube E1 dropped her side of the tube and placed her hands and face downwards for an interruption period of 15 seconds. Following the interruption period E1 looked back up, picked up the tube and continued as before. If the infant had disengaged E1 prompted the infant’s participation to continue. After the infant had retrieved the object trial four was administered.
- *Experimental trial four*: The procedure for trial three was repeated.

*Cooperation: Trampoline*

The procedure for the *Trampoline* task was very similar to the *Tubes-with-handles* task. However, in the task the infant’s goal was to bounce a toy up and down on a handheld trampoline. Importantly, due to joints on the side of the trampoline if two people did not hold it at the same time it would collapse. Therefore, to be successful on this task the infant was required to ‘work together’ and cooperate with the experimenter. For each infant the experimental procedure included a number of steps.

- *Familiarisation and demonstration*: The task began with a brief familiarisation period where the infant was shown the trampoline and encouraged to hold it on each side. After the infant was familiar with the apparatus E1 and E2 demonstrated how a toy could be made to bounce up and down by shaking the trampoline at the rim.
- *Experimental trial one*: Following the demonstration period E1 invited the infant’s participation by alternating gaze between the infant and the trampoline. If the infant was immediately successful and cooperated with the experimenter to bounce the toy on the trampoline then trial 2 was administered. However, if the infant was not successful within 30 seconds E1 and E2 carried out the demonstration phase again. Following the second demonstration the infant was encouraged to participate again this time using verbal cues such as “Come and try” and “Look, trampoline!”. If the infant was still unsuccessful the demonstration was repeated for a third time but this time E2 also encouraged the infant to stand by her and hold the trampoline together with her. If the infant was unsuccessful after three demonstrations the task was discontinued.
- *Experimental trial two*: Following trial one, E1 briefly removed the toy then after a short period placed it back on the trampoline and invited the infant’s participation again. After five seconds of play, trial three was administered. If the infant was not successful after 60 seconds, then the task was discontinued.
- *Experimental trial three*: Following trial two, E1 briefly removed the toy then after a short period placed it back on the trampoline. The infant’s participation was once again prompted by E1. However, in this trial, unlike trials one and two, when the infant picked up their side of the trampoline E1 dropped her side and placed her hands and face downwards for an interruption period of 15 seconds. Following the interruption period E1 looked back up, picked up her side, and continued as before. If the infant had disengaged, E1 prompted the infant’s participation to continue. After five seconds of play trial four was administered.
- *Experimental trial four*: The procedure for trial three was repeated.

*Coding*

The same coding schema used by Warneken and Tomasello (2006, 2007) was used to code infant’s behaviour. For each trial of the *Tubes-with-handles* and *Trampoline* task infant’s behaviour was coded according to their level of Coordination/Engagement and their behaviour during each interruption period (Tables 1 and 2).

**Table 1. Coding schema for performance on the *Cooperation: Tubes-with-handles* task**

| Coordination | |
| --- | --- |
| *Category* | ***Definition*** |
| No success (Score=0) | Tubes not opened |
| Uncoordinated (Score=1) | Success after more than 5 seconds of inappropriate actions such as standing on wrong side, letting tube drop more than once, individual play, or individual attempts |
| Coordinated (Score=2) | Success, but some inappropriate actions, but not for more than 5 seconds; releasing handle not more than once |
| Very coordinated (Score=3) | Success after immediate understanding of their role. Infant positions herself in correct location and performs the correct action without mistakes. |
| Behaviour during interruption period | |
| *Category* | ***Definition*** |
| Disengagement | Infant leaves apparatus or plays without pursuing the goal by banging the apparatus, climbing on it, etc. |
| Individual attempt | Infant attempts to retrieve the object individually (infant attempts to hold both handles or peel it open on one side) or attempts to continue the game alone. |
| Waiting | Infant remains on correct side of the apparatus, ready to perform their role |
| Re-engagement | Infant is ready to perform their role and in addition tries to re-engage E1, e.g. pushing the tube, pointing at the object and vocalising whilst looking at the partner. |

**Table 2. Coding schema for the *Cooperation: Trampoline’* task**

| Engagement | |
| --- | --- |
| *Category* | ***Definition*** |
| No success (Score=0) | Infant does not hold and lift trampoline |
| Low engagement (Score=1) | Joint play but lots of stopping and not too excited. Infant needs a lot of persuasion. |
| Medium engagement (Score=2) | Some stopping or not too excited. |
| High engagement (Score=3) | Continuous play and rather excited (placing block on trampoline; initiating play; active shaking) |
| Behaviour during interruption period | |
| *Category* | ***Definition*** |
| Disengagement | Infant leaves apparatus or plays without pursuing the goal by banging the apparatus, climbing on it, etc. |
| Individual attempt | Infant attempts to retrieve the object individually (infant attempts to hold both handles or peel it open on one side) or attempts to continue the game alone. |
| Waiting | Infant remains on correct side of the apparatus, ready to perform their role |
| Re-engagement | Infant is ready to perform their role and in addition tries to re-engage E1, e.g. pushing the tube, pointing at the object and vocalising whilst looking at the partner. |

*Pass/fail criteria and rationale*

For each infant a median Cooperation/Engagement score was calculated across the trials. In each trial ‘no success’ received a score of zero, ‘uncoordinated’/‘low engagement’ received a score of one, ‘coordinated’/‘medium engagement’ received a score of two, and ‘very coordinated’/‘high engagement’ received a score of three. Infants were coded as having passed the *Tubes-with-handles’* task if they showed at least one re-engagement attempt during interruption periods *and* their median Cooperation score was three. Similarly, infants were coded as having passed the *Trampoline* task if they made at least one re-engagement attempt during interruption trials *and* their median Engagement score was three. These criteria were decided upon for three reasons. Firstly, as highlighted in previous literature the re-engagement attempt provided the indication that the infants possessed the social cognitive understanding to form a joint goal. Secondly, in the original experimental study it was not until 24 months with the *Tubes with handles* task, and later with the *Trampoline* task, that infants were able to coordinate their actions skilfully enough to execute a joint intention reliably towards a joint goal. A median Cooperation/Engagement score of three was decided upon as this represented ‘skilful and reliable coordination’ and would therefore be in line with the ages of developmental accomplishments noted in the original literature.
