# Supplementary material for: Scaling of Early Social Cognitive Skills in Typically Developing Infants and Children with Autism Spectrum Disorder
Source: J Autism Dev Disord. 2020 Mar 18;50(11):3988–4000. doi: 10.1007/s10803-020-04449-9 (PMC7557487; doi:10.1007/s10803-020-04449-9)
Supplement: Supplementary file 2 — Supplementary file2 (DOCX 13 kb) [file 10803_2020_4449_MOESM2_ESM.docx]

**Online Resource 2**

**Study 1: Preliminary analyses**

Preliminary analyses of the *Helping*, *Communication: Point* and *Communication: Gaze* control trials were conducted to ensure that successful performance reflected an understanding of *intention,* rather than a response to low-level attentional cues.

*Helping* control trial

The *Helping* task control trials involved scenarios in which the experimenter’s behaviour was matched to that observed in the experimental trials but ‘helping’ behaviour was not required. Out of the 76 infants that passed the required object to the examiner during an experimental trial, only 3 (3.95%) also passed an object during a control trial, and only 2 (2.63%) took possession of an object before passing it to the experimenter. This indicated that the behaviour observed in experimental trials reflected an understanding of the adult’s *intention* to access the object and a motivation to ‘help’ the adult, rather than an attempt to reinstate the original context, request that the adult repeat the action, or to obtain the object primarily for themselves.

*Communication: Point* and *Communication: Gaze* control trials

Control trials in the *Communication* tasks consisted of non-communicative cues (a ‘distracted point’ and a ‘control gaze’), designed to ensure correct responses were not the result of low-level attentional cueing. For each task (*Point* and *Gaze*), paired t-tests were used to analyse the control trial performance of those who had passed the task (i.e. those who had successfully reached for the object in both experimental trials). Analyses revealed no significant difference between the number of correct and incorrect responses for either the *Communication: Point* control trials (t(57)*=*.662, *p*=.54), or the *Communication: Gaze* control trials (t(36)*=*.466, *p*=.64). These analyses indicate that the control cues had not been sufficient to direct participants’ attention to the location of the toy, and therefore that successful task completion occurred when children understood the *intention* behind the examiner’s communicative cue during the experimental trials.
